# Supplementary material for: Relative risk of end-stage renal disease requiring dialysis in treated ankylosing spondylitis patients compared with individuals without ankylosing spondylitis: A nationwide, population-based, matched-cohort study
Source: PLoS One. 2020 Apr 20;15(4):e0231458. doi: 10.1371/journal.pone.0231458 (PMC7170243; doi:10.1371/journal.pone.0231458)
Supplement: S1 Table — Additional data. (DOCX) [file pone.0231458.s003.docx]

Table A Medication in the three groups of NSAIDs

| **Group** | **NSAID** |
| --- | --- |
| **Traditional NSAIDs** | Diclofenac |
|  | Ibuprofen |
|  | Ketoprofen |
|  | Ketorolac |
|  | Mefenamic acid |
|  | Naproxen |
|  | Sulindac |
|  | Indomethacin |
|  | Piroxicam |
|  | Acemetacin |
|  | Aceclofenac |
|  | Phenylbutazone |
|  | Flurbiprofen |
| **Selective COX-2i** | Celecoxib |
|  | Rofecoxib |
|  | Etoricoxib |
|  | Parecoxib |
| **Preferential COX-2i** | Etodolac |
|  | Meloxicam |
|  | Nabumetone |
|  | Nimesulide |

Abbreviations: NSAID, non-steroidal anti-inflammatory drug; COX-2i, cyclooxygenase-2 inhibitors.

Table B Crude and multivariable-adjusted analyses of the relative risk of end-stage renal disease requiring dialysis among 37,070 AS patients compared with 370,700 matched non-AS individuals, as shown by hazard ratios with 95% confidence intervals

| **Variable** | **Crude** | **Adjusted** |
| --- | --- | --- |
|  | HR (95% CI) | HR (95% CI) |
| **AS** |  |  |
| Non-AS | Reference | Reference |
| Treated AS | 0.39 (0.29–0.51) | 0.64 (0.47–0.89) |
| **Comorbidities** |  |  |
| Diabetes mellitus |  |  |
| No | Reference | Reference |
| Yes | 16.71 (15.08–18.51) | 2.34 (2.10–2.60) |
| Hypertension |  |  |
| No | Reference | Reference |
| Yes | 11.77 (10.59–13.09) | 1.91 (1.69–2.15) |
| IgA nephropathy |  |  |
| No | Reference | Reference |
| Yes | 27.52 (20.58–36.80) | 6.28 (4.69–8.40) |
| **Frequency of serum creatinine examination during the follow-up period** |  |  |
| Number/year = 0 | Reference | Reference |
| 0 < number/year < 1 | 3.20 (2.18–4.71) | 3.30 (2.24–4.87) |
| Number/year ≥ 1 | 105.23 (75.91–145.87) | 72.56 (51.85–8102.44) |
| **NSAIDs** |  |  |
| Traditional NSAIDs |  |  |
| cDDD/year ≤ 2 | Reference | Reference |
| 2 < cDDD/year ≤ 6 | 0.50 (0.43–0.57) | 0.51 (0.45–0.59) |
| 6 < cDDD/year ≤ 14 | 0.45 (0.39–0.52) | 0.42 (0.36–0.49) |
| cDDD/year > 14 | 0.65 (0.57–0.74) | 0.45 (0.39–0.51) |
| Selective COX-2i |  |  |
| cDDD/year ≤ 8 | Reference | Reference |
| cDDD/year > 8 | 1.05 (0.86–1.28) | 0.52 (0.44–0.60) |
| Preferential COX-2i |  |  |
| cDDD/year ≤ 2 | Reference | Reference |
| cDDD/year > 2 | 1.49 (1.30–1.70) | 0.61 (0.53–0.70) |
| **Medication use vs. not use** |  |  |
| Methotrexate | 0.55 (0.30–0.99) | 0.35 (0.20–0.61) |
| Sulfasalazine | 1.61 (1.45–1.79) | 1.21 (1.08–1.35) |
| Ciclosporin | 4.15 (2.35–7.33) | 2.90 (1.60–5.25) |
| Corticosteroid | 1.61 (1.45–1.79) | 1.30 (1.16–1.45) |
| Aminoglycoside | 4.56 (3.57–5.83) | 1.30 (1.01–1.66) |
| Amphotericin B | 7.92 (3.29–19.06) | 2.14 (0.88–5.22) |
| Cisplatin | 0.34 (0.09–1.35) | 0.11 (0.03–0.45) |
| Contrast agents | 2.07 (1.73–2.48) | 0.75 (0.62–0.90) |

Abbreviations: AS, ankylosing spondylitis; HR, hazard ratio; CI, confidence interval; NSAIDs, non-steroidal anti-inflammatory drugs; cDDD, cumulative defined daily dose; COX-2i, cyclooxygenase-2 inhibitors.

Table C Multivariable-adjusted Cox regression analyses of the relative risk of end-stage renal disease requiring dialysis shown by hazard ratios with 95% confidence intervals in 37,070 AS patients compared with 370,700 matched non-AS individuals, as stratified by age and sex

| Group | HR (95% CI) | P-value for interaction |
| --- | --- | --- |
| **Age group** |  | 0.916 |
| ≤40 years | 0.31 (0.10–0.97) |  |
| >40 years | 0.61 (0.43–0.85) |  |
| **Sex** |  | 0.656 |
| Female | 0.51 (0.31–0.85) |  |
| Male | 0.66 (0.44–0.99) |  |

Matched variables included age, sex and year of the index date. Adjusted variables included diabetes, hypertension, IgA nephropathy, frequency of serum creatinine examinations during the follow-up period, average annual number of cumulative defined daily dose of non-steroidal anti-inflammatory drugs, use of methotrexate, sulfasalazine, ciclosporin aminoglycoside, amphotericin B, cisplatin and contrast agents.

Abbreviations: AS, ankylosing spondylitis; HR, hazard ratio; CI, confidence interval.

Table D Multivariable-adjusted Cox regression analyses of the relative risk of end-stage renal disease requiring dialysis shown by hazard ratios with 95% confidence intervals in 6,621 AS patients compared with 6,621 matched non-AS individuals, as stratified by age and gender

| Group | HR (95% CI) | P-value for interaction |
| --- | --- | --- |
| **Age group** |  | 0.895 |
| ≤40 years | 1.23 (0.13–11.74) |  |
| >40 years | 0.86 (0.30–2.43) |  |
| **Gender** |  | 0.656 |
| Female | 1.06 (0.17–6.71) |  |
| Male | 0.85 (0.28–2.63) |  |

Matched variables included age, sex, year of the index date and average annual number of cumulative defined daily dose of non-steroidal anti-inflammatory drugs. Adjusted variables included diabetes, hypertension, IgA nephropathy, frequency of serum creatinine examinations during the follow-up period, use of methotrexate, sulfasalazine, ciclosporin aminoglycoside, amphotericin B, cisplatin and contrast agents.

Abbreviations: AS, ankylosing spondylitis; HR, hazard ratio; CI, confidence interval.
